# Supplementary material for: Causal Relationship between Aging and Anorexia Nervosa: A White-Matter-Microstructure-Mediated Mendelian Randomization Analysis
Source: Biomedicines. 2024 Aug 16;12(8):1874. doi: 10.3390/biomedicines12081874 (PMC11351342; doi:10.3390/biomedicines12081874)
Supplement: Supplementary file 1 [file biomedicines-12-01874-s001.zip › Supplementary Figure/Figure S8/Leaveoneout plot of MR analysis for TL to ubm-b-2008.pdf]

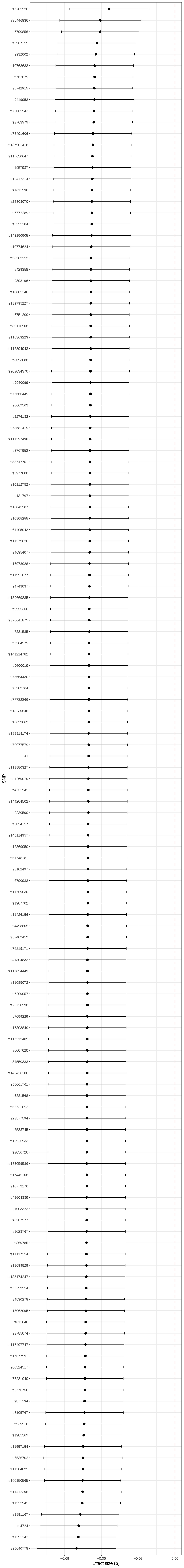
rs7705526
rs35446936
rs7790856
rs2967355
rs932002
rs10768683
rs762679
rs5742915
rs9419958
rs76065543
rs2763979
rs78491606
rs137901416
rs117630647
rs1957937
rs12412214
rs1611236
rs28363070
rs7772289
rs2555104
rs143190905
rs10774624
rs28502153
rs429358
rs9398196
rs10805346
rs139795227
rs6751209
rs80116508
rs116863223
rs112394943
rs3093888
rs202034370
rs9940099
rs76666449
rs6669563
rs2276182
rs73581419
rs111527438
rs3767952
rs55747751
rs2977608
rs10112752
rs131797
rs10845387
rs10905255
rs61405042
rs11579626
rs4695407
rs16978028
rs11991877
rs4743037
rs139669835
rs9955360
rs376641875
rs7221585
rs6584579
rs141214782
rs9600019
rs75664430
rs2282764
rs77732866
rs13230646
rs6659669
rs188918174
rs79977579
All
rs111950327
rs41269079
rs4731541
rs144204502
rs2230590
rs6054257
rs145114957
rs12369950
rs61748181
rs8102497
rs6790988
rs11769630
rs1907702
rs11426156
rs4498805
rs59409453
rs76219171
rs41304832
rs117034449
rs11085072
rs7209057
rs73730598
rs7099229
rs17803849
rs117512405
rs6007020
rs34550383
rs142426306
rs56061761
rs6881568
rs66731853
rs28577594
rs2538745
rs12925933
rs2056726
rs182059586
rs17445108
rs10773176
rs45604339
rs1003322
rs6587577
rs1023767
rs869785
rs11117354
rs11699829
rs185174247
rs56799554
rs4530278
rs13062095
rs611646
rs3785074
rs117407747
rs17677991
rs80324517
rs77231040
rs6776756
rs871134
rs8105767
rs939916
rs1985369
rs11557154
rs6536702
rs11584821
rs150150565
rs11412296
rs1332941
rs3891167
rs4724
rs1291143
rs35640778
SNP
−0.09
−0.06
−0.03
0.00
Effect size (b)
